# Genomic technologies for detecting structural variations in hematologic malignancies

**DOI:** 10.1007/s44313-024-00001-1

**Published:** 2024-02-13

**Authors:** Mi-Ae Jang

**Affiliations:** Department of Laboratory Medicine and Genetics, Samsung Medical Center, Sungkyunkwan University School of Medicine, 81 Irwon-Ro, Gangnam-Gu, Seoul, 06351 Korea

**Keywords:** Molecular diagnostics, Next-generation sequencing, Leukemia, Lymphoma, Myeloma, Myeloid, Lymphoid

## Abstract

Genomic structural variations in myeloid, lymphoid, and plasma cell neoplasms can provide key diagnostic, prognostic, and therapeutic information while elucidating the underlying disease biology. Several molecular diagnostic approaches play a central role in evaluating hematological malignancies. Traditional cytogenetic diagnostic assays, such as chromosome banding and fluorescence in situ hybridization, are essential components of the current diagnostic workup that guide clinical care for most hematologic malignancies. However, each assay has inherent limitations, including limited resolution for detecting small structural variations and low coverage, and can only detect alterations in the target regions. Recently, the rapid expansion and increasing availability of novel and comprehensive genomic technologies have led to their use in clinical laboratories for clinical management and translational research. This review aims to describe the clinical relevance of structural variations in hematologic malignancies and introduce genomic technologies that may facilitate personalized tumor characterization and treatment.

## Introduction

Hematologic malignancies are characterized by recurrent chromosomal and molecular abnormalities that are involved in disease pathogenesis and clinical heterogeneity [1, 2]. Structural variation (SV) is generally defined as a variation within a genome larger than 50 bps [3]. SVs can include a DNA region with a change in copy number (deletions, insertions, or duplications), orientation (inversions), or chromosomal location (translocations) and are often complicated by a combination of these basic changes [4]. Identifying SVs has become an essential component in the clinical management of hematologic malignancies, supporting diagnosis and prognosis, and informing therapeutic decisions [5–7].

Cytogenetics, the study of chromosome number and structure, has long been a fundamental component of diagnostic genomic analyses. A complete cytogenetic analysis of bone marrow by chromosome banding assay (CBA) should be performed at the initial evaluation and periodically thereafter to establish the cytogenetic profile and detect genetic evolution evidence [1, 8]. Fluorescence in situ hybridization (FISH) and reverse transcriptase-polymerase chain reaction (RT-PCR) are effective for identifying gene rearrangements that are not apparent in the initial CBA [9]. Depending on the abnormality, quantitative PCR and/or RT-PCR performed at the time of diagnosis can be used for minimal residual disease assessment and to monitor response to therapy [10, 11]. The use of next-generation sequencing (NGS) technologies can facilitate the sensitive and accurate detection of many common gene rearrangements and has emerged as an alternative to RT-PCR and FISH for detecting pathogenic fusion genes in hematologic malignancies [1, 10–12]. More recently, other novel genome-wide technologies, such as optical genome mapping (OGM) technology, have emerged [13–15].

This review describes a concise overview of SVs that are important for detecting several major categories of hematological malignancies, along with the key genomic technologies used for their detection.

## Key somatic SVs important for hematologic malignancy

Table 1 summarizes the key somatic SVs for the diagnostic and/or prognostic value of hematologic malignancies.

**Table 1:** Key somatic structural variations of diagnostic and/or prognostic values in selected hematologic malignancies

Disease subtype	Prognostic implication	Chromosomal/molecular abnormality
Myeloid neoplasms AML [11]	Good Intermediate Poor	t(8;21)(q22;q22.1), inv(16)(p13.1q22) or t(16;16)(p13.1;q22) t(9;11)(p21.3;q23.3) t(6;9)(p23;q34.1), t(v;11q23.3), t(9;22)(q34.1;q11.2), inv(3)(q21.3q26.2) or t(3;3)(q21.3q26.2), -5 or del(5q), -7, -17/abn(17p), complex karyotype, monosomal karyotype
MDS [16]	Very good Good Intermediate Poor Very poor	-Y, del(11q) normal karyotype, del(5q), del(12p), del(20q), double including del(5q) del(7q), + 8, + 19, i(17q), any other single or double independent clones -7, inv(3)/t(3q)/del(3q), double including -7/del(7q), complex: 3 abnormalities complex karyotype > 3 abnormalities
CML [17]	Poor	+ 8, + Ph, i(17q), + 19, -7/7q-, 11q23 or 3q26.2 aberrations, complex karyotype
Lymphoid neoplasms B-ALL [10]	Good Poor	t(12;21)(p13;q22), high hyperdiploidy (51–65 chromosomes) t(9;22)(q34.1;q11.2), hypodiploidy (≤ 45 chromosomes), iAMP 21, t(17;19)(q22;p13), t(v;11q23.3)
CLL [18]	Favorable Intermediate Unfavorable	del(13q) (as a sole abnormality) normal karyotype, + 12 del(17p), del(11q)
MM [19]	High risk	del(17p), t(4;14)(p16;q32), t(14;16)(q32;q23)
FL [20]		t(14;18)(q32;q21)
MCL [20]		t(11;14)(q13;q32)
Burkitt [20]		t(8;14)(q24;q32)

*Abbreviations*: *AML* acute myeloid leukemia, *MDS* myelodysplastic syndrome, *CML* chronic myeloid leukemia, *B-ALL* B-lymphoblastic leukemia/lymphoma, *CLL* chronic lymphocytic leukemia, *MM* multiple myeloma, *FL* follicular lymphoma, *MCL* mantle cell lymphoma, + *Ph* second or extra copy of the Ph chromosome, *iAMP21* intrachromosomal amplification of chromosome 21

### Myeloid neoplasms

Recurrent SVs in acute myeloid leukemia (AML) and myelodysplastic syndrome (MDS) are associated with distinctive clinicopathological features and have prognostic significance [1, 11, 21–23]. Those that are identified most commonly in AML are balanced SVs such as t(8;21)(q22;q22.1), inv(16)(p13.1q22) or t(16;16)(p13.1;q22), t(15;17)(q24.1;q21.2), and t(9;11)(p21.3;q23.3), which create fusion genes encoding chimeric proteins that contribute to leukemogenesis [2]. In MDS, recurrent SVs provide presumptive molecular evidence for diagnosis and have the strongest prognostic impact in the Revised International Prognostic Scoring System (R-IPSS) with cytogenetic risk classification [16]. For example, the World Health Organization (WHO) and the International Consensus Classification (ICC) have separated MDS with low blasts and isolated del(5q) into distinct subcategories, reflecting its favorable prognosis and sensitivity to lenalidomide therapy [21–23]. Chronic myeloid leukemia (CML) is characterized by the presence of t(9;22)(q34;q11.2), which generates the oncogenic tyrosine kinase *BCR::ABL1* fusion gene. In addition, the European Leukemia Net (ELN) recommends that CBA be performed at diagnosis and follow-up to detect additional chromosomal aberrations in CML, such as + 8 extra copies of the Ph-chromosome, i(17q), which may indicate disease progression and treatment failure or resistance [17].

### Lymphoid neoplasms

The genomic landscape of B-lymphoblastic leukemia/lymphoma (B-ALL) is complex and has various key driver mutations, making it one of the most challenging entities to characterize [2, 5]. SVs are detected in over 75% of B-ALL cases and can be divided into two main prognostic groups depending on whether the outcomes are good or poor, as summarized in Table 1 [5]. Despite significant advances in T-lymphoblastic leukemia/lymphoma (T-ALL), there is still insufficient evidence to establish genetically defined types of T-ALL with clinical relevance, despite significant advances in our understanding of the genetic background of the disease [24]. Chronic lymphocytic leukemia has an abnormal karyotype observed in approximately 80% of cases, with the most frequent alterations being del(13q), del(11q)/*ATM*, trisomy of 12, del(17p)/*TP53*, del(6q) and 14q32 rearrangements, and these abnormalities are associated with patient outcome [18]. The Revised International Staging System for multiple myeloma (MM) includes serum beta-2 microglobulin, serum albumin, prognostic information from serum lactate dehydrogenase, and high-risk chromosomal abnormalities [19]. Patients with MM with del(17p) and/or translocations t(4;14) and/or t(14;16) were considered at high risk, whereas those without high-risk chromosomal abnormalities were considered at standard risk [25]. Table 1 summarizes the non-Hodgkin lymphomas that are associated with specific cytogenetic abnormalities.

## Conventional cytogenetic diagnostic assays

Cytogenetic analysis of neoplastic blood- and/or bone marrow-acquired clonal chromosomal abnormalities is important for managing patients with hematologic malignancies. The analysis results can facilitate the classification of disease types, influence therapy, and prognosis, and may be used to monitor the disease course in patients. Cytogenetic analyses included CBA, FISH, and chromosomal microarray (CMA).

### Chromosome banding assay

A chromosome band can be distinguished from its adjacent segments by its darker or lighter appearance using different band-staining methods. G-banding is the most common technique used in CBAs and uses a Giemsa dye mixture to produce a visible karyotype. This resulted in a nearly constant pattern of dark and light bands along chromosomes. CBA should be performed on living cells that are still dividing or can be stimulated to divide again, with cell culture conditions optimized for suspected hematologic malignancies [8]. For complete CBA, a sufficient number of analyzable metaphase cells (a minimum of 20 cells) must be obtained during cell culture [8].

CBA provides whole genome information, allowing screening for both numerical (loss or gain of a whole chromosome) and gross structural (translocations, deletions, and inversions) abnormalities that occur in most hematologic malignancies at a single cell level [5, 6]. The distinction between individual cell clones allows the differentiation between primary and secondary chromosomal abnormalities; thus, clonal evolution can be inferred from the karyotype [5]. Currently, for most hematologic malignancies, CBA is an essential diagnostic workup component, guiding the diagnosis of genetic subtyping of the disease supported by the WHO classification [21, 24] and ICC [22, 26], and providing clinical care supported by the ELN [27], National Comprehensive Cancer Network [10, 11, 20, 23, 28], and R-IPSS [29].

### Fluorescene in situ hybridization

FISH is a molecular diagnostic technique that uses fluorochrome-labeled probes to detect genetic or chromosome abnormalities [9]. The probe comprised DNA, typically 10–100 kb in length, hybridized to its complementary DNA sequence on chromosomal preparations previously fixed in situ on microscope slides [9]. The probe signal can be visualized using a fluorescence microscope, and chromosomal abnormalities can be detected and quantified in samples by counting and scoring the presence or absence of signals or structural abnormalities in numerous cells (usually 200–500).

FISH can be used to map loci on specific chromosomes and its sensitivity can detect structural chromosomal rearrangements and numerical abnormalities [5, 6, 30]. They can be used to identify entire chromosomes using whole-chromosome painting probes, which can help identify karyotypes with complex translocations or marker chromosomes [5]. The major advantage of FISH is that it can be performed on non-dividing interphase cells and numerous cells can be easily scored. FISH is a rapid, simple, and quantitative method with excellent probe stability [9]. Although CBA plays a limited role in detecting early relapse or minimal residual disease, FISH can be a useful tool for monitoring the remission status when clonal chromosomal abnormalities have been identified at diagnosis [5, 8].

### Chromosomal microarray

A CMA comprises thousands or millions of unlabeled DNA probes fixed to glass in a high-density grid format. A test sample containing a heterogeneous collection of labeled DNA fragments was denatured and hybridized with numerous probes on the array. The hybridization signals are detected by laser scanning, providing high-resolution copy number variation (CNV) data that can detect small abnormalities of under 1 kb across the genome [5, 6]. Microarray analysis can be performed on direct (uncultured) specimens without the need for dividing the cells, providing a more accurate assessment of abnormalities and tumor burden [5].

CMA with single-nucleotide polymorphism platforms detects allele-imbalanced regions with copy-neutral loss of heterozygosity (CN-LOH) [6]. CN-LOH has been identified as a major mechanism for tumor suppressor gene inactivation and is a frequent oncogenic event [8, 12, 31, 32]. CN-LOH has been observed in 20–46% of patients with MDS and is believed to occur through various mechanisms, including gene conversion, somatic recombination, loss of one allele, and subsequent reduplication [32].

## Limitations of conventional cytogenetic assays

Cytogenetic techniques, including CBA, FISH, and CMA, are complementary and each has specific limitations; therefore, one approach should not replace the other. An intrinsic limitation of CBA is that CBA be performed on dividing cells in vitro. In some hematological malignancies, a sufficient number of analyzable metaphase cells may not be obtained in culture for complete chromosomal analysis. Moreover, even when cells divide rapidly and sufficient metaphase is achieved, CBA has a limited resolution (approximately 10 Mb) that depends on the banding pattern, making it difficult to identify cryptic aberrations [5, 6]. Subsequently, complementary technologies such as FISH and CMA have been developed to overcome many of the main CBA limitations. These platforms do not require dividing cells and can detect abnormalities with greater sensitivity, effectively expanding the resolution from large chromosomal bands to gene-level imbalances. However, FISH using commonly used locus-specific DNA probes cannot provide genome-wide information because genetic changes are limited to specific chromosomal regions where the probes are localized [6]. In addition, FISH can only detect abnormalities larger than the probes used; therefore, deletions smaller than 50 kb may not be detected by FISH. Therefore, FISH is not an effective screening method for detecting chromosomal abnormalities. The usefulness of CMA technology is primarily limited to diseases driven by CNVs or unbalanced SVs because it is unable to detect balanced chromosomal rearrangements, which are hallmarks of many hematologic malignancies [5, 6]. Despite the widespread CBA and FISH use in clinical laboratories, the worldwide adoption of CMA has been hampered by high costs and reimbursement policies [6].

## Advances in genomic technologies

Advances in sequencing technology have made it possible to search for SVs at high resolution and explore a more comprehensive view of the genome, including single nucleotide variants (SNVs) such as *TP53*, *RUNX1*, *IDH1*, *IDH2*, *NPM1*, and *FLT3*-ITD. More recently, novel genomic technologies that provide SV information, such as OGM, have emerged. Table 2 lists the currently available genomic technologies, along with their advantages and limitations.

**Table 2:** Comparative characteristics of available genomic technologies for hematologic malignancies

Method	CBA	FISH	CMA	RT-PCR	Targeted sequencing		WGS	WTS	OGM
Analyte	Living cells	DNA in interphase and metaphase	DNA	RNA	DNA	RNA	DNA	RNA	DNA
Coverage	Genome-wide	Targeted	Genome-wide	Targeted	Targeted	Targeted	Genome-wide	Genome-wide	Genome-wide
Individual cell clone identification	Yes	Yes	No	No	No	No	No	No	No
Ability to multiplex	Low	Low	High	High	High	High	High	High	Low to medium
Resolution	> 5–10 Mb	> 100 Kb	> 15 Kb	NA	Single base	Single base	Single base	Single base	0.5–5 kb (for SV), 500 kb (for CNV)
Detection range									
SVs	Yes	Yes	No	Limited	Limited	Yes	Yes	Yes	Yes
CNVs	Yes	Yes	Yes	Limited	Limited	Limited	Yes	Limited	Yes
SNVs	No	No	No	No	Yes	Yes	Yes	Yes	No

*Abbreviations*: *CBA* chromosome banding analysis, *FISH* fluorescence in situ hybridization, *CMA* chromosomal microarray, *RT-PCR* reverse transcriptase *PCR*, *WGS* whole genome sequencing, *WTS* whole transcriptome sequencing, *OGM* optical genomic mapping, *NA* not available, *SV* structural variation, *CNV* copy number variation, *SNV* single-nucleotide variant

### Targeted sequencing

NGS can be designed to target a selected gene panel, the exome (all known genes, approximately 1–2% of the genome), or the entire genome. Gene panels target curated sets of genes associated with specific clinical phenotypes such as acute leukemia, MDS, and lymphoma. Sequencing aims to identify DNA SNVs that are confidently associated with the presenting disease [33]. NGS data allowed the detection of CNVs from targeted gene panels. This was achieved by analyzing the coverage depth of the captured regions and calculating the copy number ratio score for each region [34]. Using NGS for CNV detection will help clinical diagnostic laboratories test numerous genes for CNVs and its application requires high-quality data with consistency across regions [35].

Targeted RNA-sequencing panels have been developed to identify expressed gene fusions [36, 37]. Conventionally, the presence of gene fusions is assessed using cytogenetic assays and RNA-based RT-PCR tests. Although RT-PCR has excellent sensitivity, it requires specific primer sets for each fusion transcript; therefore, gene fusion with unusual partner genes or breakpoints may lead to false-negative results. NGS-based targeted RNA-sequencing panels overcome the limitations of conventional methods by generating numerous sequencing reads in parallel using numerous probes and primers targeting expanded genes and regions [38]. Therefore, it is an efficient method for detecting gene fusions in various hematologic malignancies [36, 38–40]. NGS can be applied in comprehensive assays to identify gene mutations, fusions, and expression by simultaneously assessing DNA and RNA in a single step [41–43].

### Whole genome sequencing

Whole-genome sequencing (WGS) analyzes the DNA sequence of the entire genome and provides an unbiased and comprehensive overview of all three genomic variants: SNVs, CNVs, and SVs [44–46]. Mate-pair sequencing is a variation of WGS that uses specialized library preparation of long input DNA (2–5 kb), circularized and fragmented into smaller paired-end fragments (200–500 bp), and then sequenced at a reduced depth [47–49]. This assay was designed to detect SVs and CNVs throughout the genome, resulting in a cost-effective strategy that is suitable for clinical diagnostic laboratories. A recent study by Duncavage et al*.* proposed a paradigm shift in clinical laboratory testing by suggesting WGS as an alternative to cytogenetic analysis of myeloid cancers [50]. We performed WGS on a cohort of 263 patients with AML or MDS and confirmed 40 recurrent translocations and 91 CNVs previously identified in cytogenetic studies [50]. In addition, WGS identified additional genomic events of CNVs and/or SVs in 17% (40/235) of patients [50]. Ryan et al. detected 294 subtype-defining genetic abnormalities in 96% (202/210) of the study patients (210 childhood B-ALL) [44]. Despite these advantages, the clinical adoption of WGS is limited worldwide because of the complexity of its processes and workflows and the resources necessary for its implementation compared to other technologies [6].

### Whole transcriptome sequencing

Whole transcriptome sequencing (WTS) is a method that can detect gene fusions by analyzing the sequences of all expressed genes, which may improve hematologic malignancy classification and risk stratification [51]. The WTS has shown promising results in ALL [52], AML [53], and MM [54]. A key advantage of WTS in a clinical diagnostic setting is its ability to target gene fusions, even those that may be missed or cryptic when tested using conventional diagnostic approaches [51]. WTS can be used for genomic classification to diagnose known and novel oncogenic drivers and molecular subtypes of leukemia, such as Ph-like ALL, using gene expression profiles [52]. Although targeted RNA sequencing panels are commonly used in hematological malignancies, WTS has not been widely adopted in clinical settings [6].

### Optical genome mapping

OGM is a new genomic technology that can reveal SVs, CNVs, and whole-chromosome aneuploidies in a single experiment [13]. OGM electrophoreses ultra-long high-molecular-weight DNA (> 150 kb) into nanochannels, linearizing them for imaging, and creates a consensus genome map from the processed images [13]. Recent studies have highlighted the advantages of OGM as an emerging genomic technology for hematologic malignancies, which can potentially replace conventional cytogenetic diagnostics [13–15, 55–60]. In 2021, Neveling et al*.* compared OGM with CBA, FISH, and CMA in 52 samples from various hematological malignancies, including AML, MDS, chronic lymphocytic leukemia, CML, and lymphoma [15]. They reported that OGM detected variants described by conventional diagnostics in 96% (50/52) of cases. Similar studies have reported high concordance with standard diagnostics (88–95%) and the ability to obtain additional cytogenetic information missed by routine work-ups in a significant number of patients (13–64%) [55–60].

However, OGM has some limitations. They cannot detect SVs located within repeated sequences, such as centromeres or telomeres [14]. Additionally, it cannot observe ploidy changes because the software calculates the relative number of gene copies, making it prone to errors if the entire genome is hyperdiploid or hypodiploid [14].

## Conclusion

Genomic technologies are an integral part of the current clinical management of hematological malignancies. The diagnostic assessment of hematologic malignancies requires the detection of various types of genomic alterations, including SNVs, insertions and deletions, oncogenic fusions, SVs, and CNVs. Thus, the evaluation of many hematological malignancies currently requires the use of various testing methods, including CBA, FISH, CMA, targeted NGS, and RT-PCR, to detect clinically relevant genomic alterations. While no single test can replace the currently available ones, it is important that our laboratory and clinical community actively explore and synthesize a more dynamic response to advances emerging from new and comprehensive genomic technologies and update guidelines effectively.
